# High-Resolution Temperature Sensor Based on Single-Frequency Ring Fiber Laser via Optical Heterodyne Spectroscopy Technology

**DOI:** 10.3390/s18103245

**Published:** 2018-09-27

**Authors:** Liangcheng Duan, Haiwei Zhang, Wei Shi, Xianchao Yang, Ying Lu, Jianquan Yao

**Affiliations:** 1 Institute of Laser and Optoelectronics, Key Laboratory of Optoelectronics Information Science and Technology (Ministry of Education), School of Precision Instrument and Optoelectronics Engineering, Tianjin University, Tianjin 300072, China; dlc@tju.edu.cn (L.D.); yangxianchao@tju.edu.cn (X.Y.); luying@tju.edu.cn (Y.L.); jqyao@tju.edu.cn (J.Y.); 2 Tianjin Key Laboratory of Film Electronic and Communication Device, College of Electrical and Electronic Engineering, Tianjin University of Technology, Tianjin 300384, China

**Keywords:** fiber optics sensors, high resolution, single-frequency laser, heterodyne spectroscopy

## Abstract

We demonstrate a high-resolution temperature sensor based on optical heterodyne spectroscopy technology by virtue of the narrow linewidth characteristic of a single-frequency fiber laser. When the single-frequency ring fiber laser has a Lorentzian-linewidth <1 kHz and the temperature sensor operates in the range of 3−85 °C, an average sensitivity of 14.74 pm/°C is obtained by an optical spectrum analyzer. Furthermore, a resolution as high as ~5 × 10^−3^ °C is demonstrated through optical heterodyne spectroscopy technology by an electrical spectrum analyzer in the range of 18.26–18.71 °C with the figure of merit up to 3.1 × 10^5^ in the experiment.

## 1. Introduction

Optical fiber sensors have been widely investigated due to their attractive properties of anti-electromagnetic interference, high sensitivity and the facilitation of long-distance sensing. For the past few decades, various fiber structures, such as fiber Bragg grating (FBG), long period grating (LPG), Fabry-Perot interferometer (FPI), photonic crystal fiber (PCF) and Sagnac interferometer (SI), have been developed for sensing parameters, including temperature, strain, refractive index (RI) and so on [1,2,3,4,5,6,7].

Considering the different sensing architectures, performance improvement of the sensitivity and resolution of fiber-optic temperature sensors is one of the research directions that has been given extensive attention. By virtue of tracing the resonant wavelength between the fundamental core mode of PCF and the fundamental mode of liquid rod, the experimental temperature sensitivity of 54.3 nm/°C was obtained by selectively filling the 1.46-RI liquid into one of the air holes of PCF [1]. The figure of merit (FOM) that was defined as the ratio between the sensitivity and the full width half maximum (FWHM) is often used to describe the overall performance of fiber sensors [2]. Though the sensor that was based on PCF had a high sensitivity, its broad transmission-dip (~40 nm) made its sensing accuracy compromised. Therefore, optical spectrum with a narrow linewidth is indispensable for a high-resolution sensor, where spectrum filters such as FPI and FBG are generally employed. For example, a micro-fabricated fiber-optic Fabry-Perot (FP) sensor has been developed to achieve a temperature resolution of 6 × 10^−4^ °C by the average wavelength tracking method [3]. However, the fabrication technique of the FP cavity in the micron dimension, in addition to complicated data processing, constrained the practical application. On the other hand, the π-phase-shift FBG with an ultra-narrow bandwidth in the MHz level provided another routine to realize high-resolution temperature sensors, of which up to 1.4 × 10^−3^ °C was obtained with the double-sideband interrogation method and the cross-feedback technique was employed to alleviate the measurement error in the birefringence-introduced frequency difference [4].

All of the demonstrations summarized above are based on passive filtering of an external light source, and sensing information was then encoded in wavelength. Compared to that, the active sensing method, where the sensing structure acts as both the sensing element and the wavelength filter of an optical fiber laser, was proposed and has attracted intensive attention recently. Sensors based on optical fiber laser have been demonstrated to possess a narrower spectral width and a higher signal-to-noise ratio (SNR), which immensely improves the sensing performance by increasing the FOM of the sensing system. Therefore, it could meet the need on high resolution much better for applications of structural health monitoring, industrial process control and oceanographic monitoring [8,9,10]. More specifically, a robust thermometer with a high accuracy of 0.001 °C or better is necessary in quantifying thermal structures in the ocean [10]. Such a high accuracy is also critical in understanding the physical processes that are associated with mixing events, especially those that happen rapidly on small scales.

Single-frequency fiber laser (SFFL), which obtains a laser linewidth in the level of several kilohertz or even hundreds of hertz, can be a good candidate for the applications on high-resolution fiber-optic sensors. For the development of SFFL in the eye-safe wavelength regime of around 1.5 µm, inherent advantages have been found and have been employed to improve the resolution for spectroscopy, coherent light detection as well as free-space optical communications [11,12,13]. Therefore, it is feasible to realize an active intra-cavity temperature sensor with performance improvement on wavelength-interrogation precision and resolution.

For an optical fiber sensor that is based on SFFL with such a narrow linewidth, tiny wavelength shift, or more specifically, frequency shift cannot be resolved with the conventional optical spectrum analyzer (OSA) or scanning FPI, which have a resolution that is far big enough to make full use of the benefits of a narrow linewidth. Therefore, measuring the shift of the beat frequency between the eigen-polarization modes of a SFFL itself [14] or between the SFFL under test and an external narrow-linewidth fiber laser [15] is an effective way to exploit the characteristic of a narrow-linewidth SFFL. A temperature sensor that is based on dual-polarization single-frequency distributed feedback (DFB) fiber laser was reported to achieve a temperature resolution of 0.04 °C, through measuring both the polarization beat frequency and the absolute wavelength of one polarization [16]. Another high-resolution temperature sensor was demonstrated under a similar mechanism, where a polarimetric single-frequency distributed Bragg reflector (DBR) fiber laser was employed to achieve a temperature resolution of 0.05 °C [17]. However, further improvement on temperature resolution was constrained by limited beat-frequency shift for the longitudinal mode in different polarization states. Recently, a high-resolution static strain sensor that was based on single-frequency DFB fiber laser was reported with optical heterodyne spectroscopy technology, in which a reference laser with fixed-frequency was applied to interrogate the sensor [15]. It offers a promising solution for higher sensitivity and resolution, while the thermal effects in the phase-shifted FBG-based cavity have the potential problems of grating dephasing when they are employed in temperature sensing [18].

In this paper, we experimentally demonstrate a high-resolution temperature sensor that is based on a single-frequency ring fiber laser via the optical heterodyne scheme. By using an unpumped Er-doped fiber as the saturable-absorber tracking narrow-band filter [19,20], a tunable SFFL with a linewidth of ~520 Hz was achieved in our experimental scheme. When the SFFL works as a temperature sensor over the range of 3–85 °C, the corresponding sensitivity is 14.74 pm/°C according to the relationship between the temperature of FBG and the output wavelength. When it comes to the frequency domain, a sensitivity of 1832 MHz/°C is obtained with an electrical spectrum analyzer (ESA). Assuming that the frequency stability of the heterodyne spectrum is ~10 kHz, the limiting resolution of the investigated temperature sensor is calculated and analyzed, which, theoretically, can be up to ~5 × 10^−6^ °C. However, the experimental resolution turned out to be ~5 × 10^−3^ °C due to the beat-frequency jitter that is in a level of ~9.4 MHz around the central frequency. It is also worth mentioning that the FOM of this sensing system has been increased about four orders when compared to passive sensors, which benefits from the ultra-narrow linewidth of the heterodyne spectrum.

## 2. Experimental Setup and Sensing Principle

**Figure 1:**
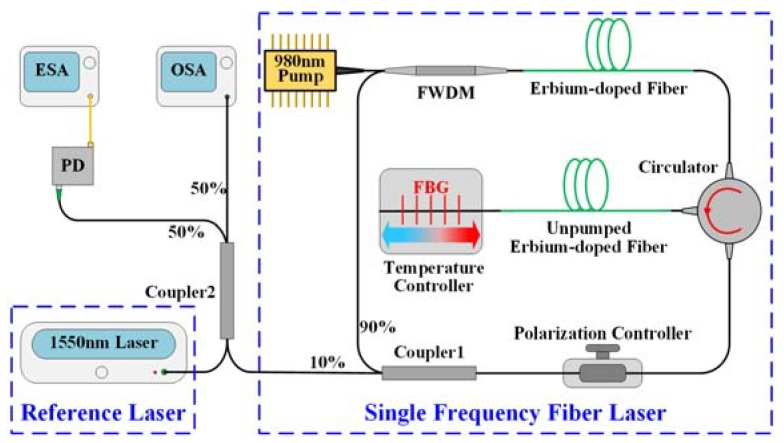
Experimental setup of the temperature sensor.

The experimental setup of the demonstrated high-resolution temperature sensor is illustrated in Figure 1. A filter wavelength division multiplexer (FWDM) is used to couple the pump light into a 2-m-long Er-doped fiber that works as a gain medium. Then, it is connected to an optical circulator so as to make the ring fiber laser operate unidirectionally and to prevent the spatial hole-burning effect. The second port of the circulator is spliced to a 3-m-long unpumped Er-doped fiber followed by a FBG centered at 1550 nm. The FBG is chosen to have a reflectivity of 70% and a 3-dB bandwidth of 0.03 nm. It is fixed on a temperature controller (TC) to tune the lasing wavelength. A polarization controller is spliced between the output coupler and the third port of the circulator to optimize the output mode. Lastly, the 10%-port of the output coupler is used to extract the light from the ring cavity.

**Figure 2:**
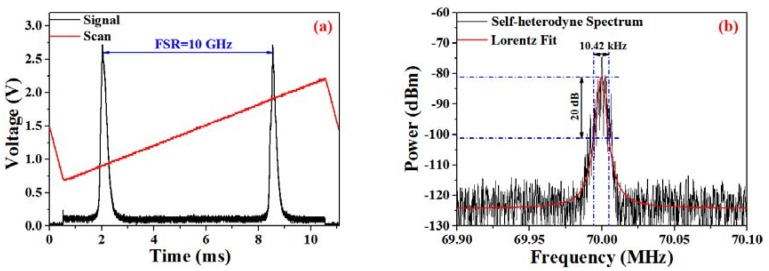
(**a**) Scanning FPI verifying single-frequency operation spectrum; (**b**) Measured and fitted lineshape of the self-heterodyne signal.

In order to obtain a single-longitudinal-mode laser output, a 3-m-long unpumped Er-doped fiber is employed to form a saturable-absorber tracking filter for linewidth narrowing. According to the spectrum that was achieved from the scanning FPI, single-longitudinal-mode operation is confirmed, as illustrated in Figure 2a. The laser linewidth is measured via the delayed self-heterodyne method [21], and the power spectral density of the self-heterodyne spectrum is depicted in Figure 2b. Considering that a 20-dB linewidth of ~10.42 kHz is achieved from the Lorentzian-fitted profile, this indicates that the laser linewidth is around 520 Hz.

**Figure 3:**
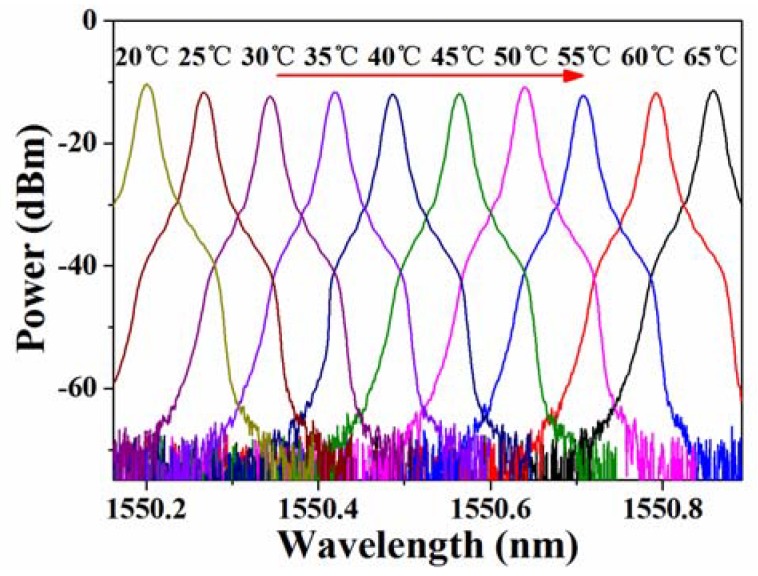
Output spectra of the single-frequency fiber laser at different temperatures.

As shown in Figure 3, the SNR of the SFFL is higher than 50 dB and the central wavelength can be tuned through changing the temperature of the FBG by fixing it in the copper groove of TC and covering it with thermal insulation material. Both the accuracy and stability of TC are 0.1 °C and it can heat or cool FBG to a desired temperature from −10 °C to 90 °C through controlling the current direction of the thermoelectric cooler (TEC) while the OSA is used to test the temperature sensor cursorily with a low resolution. Because of the one-to-one correspondence between the temperature of the FBG and the output wavelength, the tunable laser, to a certain extent, can be regarded as a sensor so as to monitor the temperature fluctuation.

**Figure 4:**
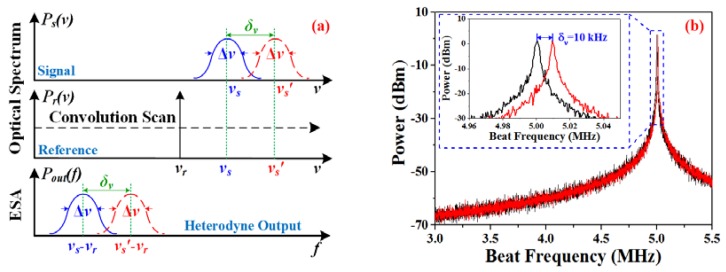
(**a**) Convolution of a narrow linewidth laser spectra translated to low frequencies; (**b**) Simulated heterodyne spectrum with a frequency shift of 10 kHz, corresponding to a temperature change of 5 × 10^−6^ °C.

Although the output laser possesses a linewidth that is less than 1 kHz, the resolution of the OSA can only be up to the order of the picometer, which is not high enough to distinguish the tiny wavelength change of the SFFL with a linewidth at the sub-kilohertz level. To make full use of the narrow-linewidth characteristic of the SFFL, the optical heterodyne system was established. As per the experimental setup that is depicted in Figure 1, a commercial tunable 1550-nm SFFL (NKT Photonics, Birkerød, Denmark, Koheras AdjustiK C15) possessing a linewidth of ~6 kHz is employed as the reference laser. The sensing SFFL and the reference laser are combined and then split into two paths via a 2 × 2 3-dB coupler. One path is directly coupled into an OSA (Yokogawa, Tokyo, Japan, AQ6370D) with a resolution of 0.02 nm to monitor the wavelength shift. The other path is sent into an ESA (Agilent, Santa Clara, CA, USA, N9030A PXA) through a high-speed photodetector (PD) (Thorlabs, Newtown, NJ, USA, DET01CFC). It is well known that the heterodyne spectral density is the convolution of the reference laser and the local SFFL [22]. By tuning the wavelength of the reference laser appropriately, the heterodyne spectra will be translated into low frequencies and its spectral density can be displayed on the ESA as it is shown in Figure 4a.

If the central wavelength of the reference laser is fixed and the temperature is changed, we can get the relationship between the temperature fluctuation and frequency shift *δ_ν_* of the SFFL by tracing the heterodyne spectra in different temperatures. Generally, the temperature drift Δ*T* is expected to have a linear relationship with the frequency shift *δ_ν_* as [23]

(1) $$\Delta T=\frac{\delta_{\nu }}{\left(\alpha +\xi \right)\cdot \nu },$$

where *α* is the thermal expansion coefficient of the FBG, which is ~1.1 × 10^−6^/°C. *ξ* denotes the thermo-optic coefficient, which has the value of ~8.3 × 10^−6^/°C, *ν* is the central frequency of the SFFL. For clarity, we also simulated the heterodyne spectra with MATLAB. As depicted in Figure 4b, the maximum resolution is determined by the linewidth and the beat-frequency stability. Therefore, according to Equation (1), the temperature sensor can be effective even with a thermal drift as small as ~5 × 10^−6^ °C, assuming that the SFFL operates around 1550 nm, the heterodyne spectrum has a linewidth of 1 kHz and the beat-frequency stability is ~10 kHz. Moreover, according to [2], the figure of merit (FOM) is defined as:

(2) $$\mathrm{FOM}=\frac{S_{\lambda }}{\mathrm{FWHM}},$$

where *S_λ_* is the sensitivity of the sensor and FWHM is the full width half maximum of the sensing spectrum. Therefore, assuming that the sensitivity is the same, such a narrow-linewidth heterodyne spectrum can also enhance the value of the FOM about five orders of magnitude compared to conventional sensing spectrum with a picometer linewidth.

## 3. Results and Discussion

To verify the relationship between the temperature and the beat frequency, we immersed the FBG in water with a digital probe thermometer inside (accuracy of 0.01 °C) to confirm the temperature around the FBG. In the experiment, the ambient temperature was about 23 °C and the initial temperature of the water was 16 °C. We measured the data when the water temperature increased slowly and constantly through heat exchange from 18.26 °C to 18.71 °C.

First of all, we made the beat frequency lie in the bandwidth of the PD by adjusting the central wavelength of the tunable reference laser. And then, the output of the reference laser was fixed to the certain wavelength.

**Figure 5:**
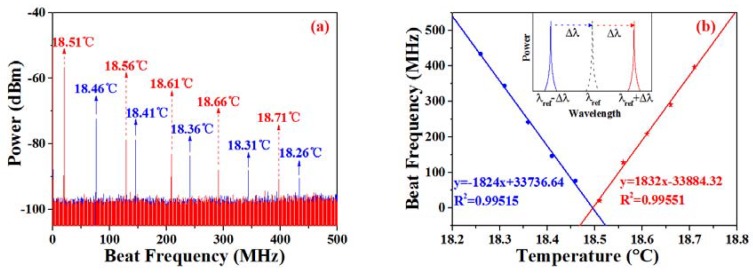
(**a**) Beat frequency spectra with the temperature increased from 18.26 °C to 18.71 °C; (**b**) Relationship between temperature and beat frequency.

As the heterodyne spectra under different temperatures shown in Figure 5a, when the water temperature rises with the ambient temperature, the central frequency of the heterodyne spectrum decreases at first, which means that the central wavelength of the SFFL approaches the reference laser constantly, as shown in the inset in Figure 5b. By recording the central frequencies of the heterodyne spectra with an interval of 0.05 °C from 18.26 °C to 18.46 °C, we found that the beat frequency decreases linearly as a function of the temperature. With the continued growth of the water temperature, the central frequency of the heterodyne spectrum, on the contrary, shifts to the higher frequencies, as it is displayed in Figure 5a. As a result, the beat frequency begins to increase linearly when the water temperature changes over a range from 18.51 °C to 18.71 °C. According to the best-fitting straight line that is depicted in Figure 5b, the two lines almost have the same slope efficiency, which indicates that the SFFL functions well as a temperature sensor in the range of 18.26–18.71 °C. The maximum slope efficiency is 1832 MHz/°C, corresponding to the maximum sensitivity of 14.65 pm/°C around 1550.20 nm. Though the sensitivity is lower than the temperature sensor realized via PCF, the linewidth of the heterodyne spectrum is extremely narrow, leading to the FOM (~3.1 × 10^5^) of our sensing system improving by five orders of magnitude when compared with [1].

**Figure 6:**
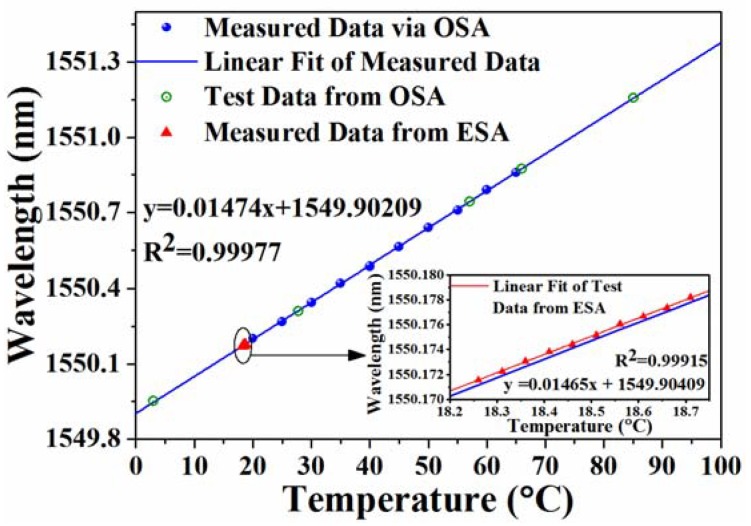
Relationship between the temperature and the wavelength of the single-frequency fiber laser.

It should be mentioned that the temperature detection range is limited by the bandwidth of the PD. It can be twice of the detector bandwidth in our experiment, as shown in Figure 5. Moreover, the detection range can be further broadened by changing the operating wavelength of the reference laser. Due to the lack of broader bandwidth PD, we investigated the compatibility of the temperature sensor in a larger detection range by employing the OSA with a resolution of 0.02 nm as the demodulation equipment. When the temperature changes from 20 °C to 65 °C with an interval of 5 °C, the central wavelength increased from 1550.36 nm to 1550.89 nm, as it is shown in Figure 6. The best-fitting straight line indicates that the sensitivity is about 14.74 pm/°C. Then, we sampled some points at random to test the sensing accuracy and repeatability. As the test data illustrates in Figure 6, all of the sample points are located in the best-fitting line. This indicates that the sensor can realize stable operation with linearity in the temperature range of 3–85 °C. To further verify the accuracy of the optical heterodyne method, all of the data that was measured from ESA were converted from frequency domain to wavelength domain and were added to Figure 6 with red triangle symbols. According to the inset with a zoomed temperature range in Figure 6, it coincides well with the linear fit of the measured data via OSA. The maximum deviation between the two fitting lines is only ~0.5 pm, which was mainly induced by the different resolution between the OSA and the optical heterodyne method.

**Figure 7:**
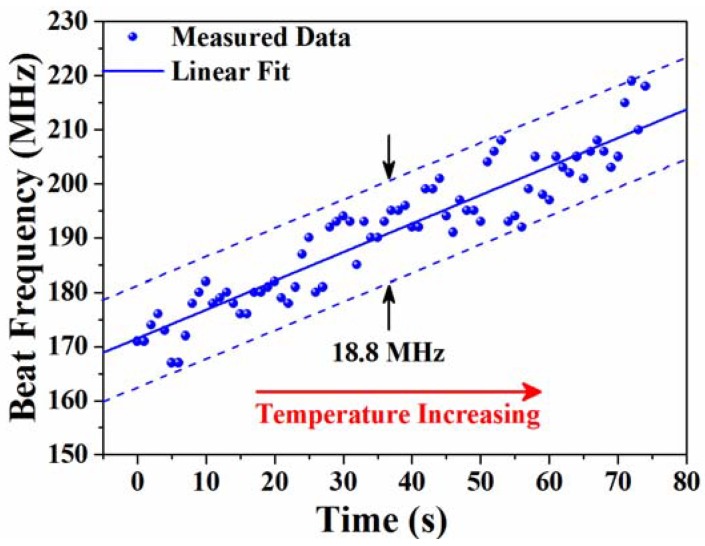
Beat-frequency stability of the heterodyne spectrum.

It is indicated in Figure 4b that the temperature resolution is not only related to the laser linewidth, however it is also affected by the frequency stability of the heterodyne spectrum. Therefore, it is necessary to characterize the beat-frequency stability for the actual temperature resolution of our experiment setup. Since the beat frequency is very sensitive to the temperature change and it is difficult to keep the temperature constant, we set up the temperature of the water as lower than the ambient temperature and measured the frequency fluctuation during natural water temperature increases. As per the results that are depicted in Figure 7, the maximum beat-frequency jitter around the central frequency is about 9.4 MHz, which means that a temperature resolution of up to ~5 × 10^−3^ °C can be achieved in our experimental scheme according to Equation (1).

For clarity, performance comparisons of the proposed sensor with the sensors that were referred to prior are listed in Table 1. As we mentioned in the introduction, for passive sensing approaches, although relatively high sensitivities or resolutions are obtained, their practical applications are limited by relatively broader FWHM, complex micro-fabrication of the sensing head, complicated data processing and lower SNR. As for active sensing approaches, sensing heads are often commercially available FBGs, and narrower linewidth and higher SNR are easier to achieve, making them more suitable for long distance sensing and a potential for extremely high-resolution sensors with a higher FOM.

## 4. Conclusions

A high-resolution temperature sensor that was based on optical heterodyne spectroscopy technology was investigated and was experimentally demonstrated in this work. When the temperature of the FBG in the SFFL changed in the range from 3 °C to 85 °C, a sensitivity of 14.74 pm/°C was achieved by measuring the output wavelength shift. Through further monitoring the beat frequency between the self-made SFFL and the reference fiber laser, a resolution up to ~5 × 10^−3^ °C was obtained in our demonstration. Considering that the resolution was limited by the beat-frequency stability, the theoretical resolution of the investigated temperature sensor could be up to ~5 × 10^−6^ °C if the frequency stability of the heterodyne spectrum could be promoted to 10 kHz. It can be anticipated that the sensitivity improvement can be further improved by using FBG written in the material with a high thermal-optic coefficient. What is more, the system we demonstrated here with a narrow-linewidth single-frequency fiber laser can also be employed for high-resolution sensing on other parameters, such as strain, RI and pressure.

## Figures and Tables

**Table 1:** Performance comparisons of the proposed sensor with sensors referred to prior.

Ref.	Sensing Approach	Sensitivity	Resolution (°C)	Dynamic Range (°C)	FWHM	FOM
[1]	PCF, passive sensing	54.3 nm/°C	0.027 (calculated)	34–35.5	40 nm	1.36
[3]	FPI, passive sensing	84.6 pm/°C	0.0006	20–100	-	-
[4]	FBG, passive sensing	1228.4 MHz/°C	0.0014	19–21	40 MHz	30.71
[16]	FBG, active sensing	(1.623 ± 0.002) MHz/°C	0.04	15–45	10 kHz	162.5
[17]	FBG, active sensing	1.32 MHz/°C	0.05	10–52	-	-
This paper	FBG, active sensing	1832 MHz /°C	0.005	3–85	6 kHz	3.1 × 10^5^
